# Magnetic Hyperthermia on γ-Fe_2_O_3_@SiO_2_ Core-Shell Nanoparticles for mi-RNA 122 Detection

**DOI:** 10.3390/nano11010149

**Published:** 2021-01-09

**Authors:** Marie-Charlotte Horny, Jean Gamby, Vincent Dupuis, Jean-Michel Siaugue

**Affiliations:** 1 Physico-chimie des Électrolytes et Nanosystèmes Interfaciaux, PHENIX, Sorbonne Université, CNRS, F-75005 Paris, France; mcha.horny@gmail.com (M.-C.H.); vincent.dupuis@sorbonne-universite.fr (V.D.); 2 Centre de Nanosciences et de Nanotechnologies, Université Paris-Saclay, CNRS, 91120 Palaiseau, France; 3 Laboratoire Interfaces et Systèmes Electrochimiques, LISE, Sorbonne Université, CNRS, F-75005 Paris, France

**Keywords:** nucleic acids, magnetic hyperthermia, core-shell nanoparticles, early diagnosis

## Abstract

Magnetic hyperthermia on core-shell nanoparticles bears promising achievements, especially in biomedical applications. Here, thanks to magnetic hyperthermia, γ-Fe_2_O_3_ cores are able to release a DNA target mimicking the liver specific oncotarget miRNA-122. Our silica coated magnetic nanoparticles not only allow the grafting at their surface of a significant number of oligonucleotides but are also shown to be as efficient, by local heating, as 95 °C global heating when submitted to an alternative magnetic field, while keeping the solution at 28 °C, crucial for biological media and energy efficiency. Moreover, a slight modification of the silica coating process revealed an increased heating power, well adapted for the release of small oligonucleotides such as microRNA.

## 1. Introduction

Magnetic nanoparticles (MNP) under a high frequency alternative magnetic field (AMF) exhibit an outstanding heating behavior also referred as magnetic hyperthermia. The coupling of their magnetic dipole moments with the AMF yields the thermal losses linked to the magnetic relaxation process of the particle [1,2]. By heating iron oxide nanoparticles functionalized with DNA under an AMF, Dias et al. assessed the local temperature increase in the vicinity of a magnetic nanoparticle. They established an increase of 8 °C compared to the bulk solution at 5 nm from the nanoparticle surface [3]. This increase in temperature at the nanoscale is the key of many applications in cancer treatment [4,5] using thermosensitive drug delivery systems for targeted therapy [6]. Indeed, the global heating of a biological solution is a tricky issue in an amplification and/or detection of a biological target. Beside an obvious denaturation of the parts non compatible with high temperatures, a feedback loop is needed to take into account the thermal losses and precisely tune the temperature [7]. Magnetic hyperthermia on dilute assemblies of maghemite nanoparticles, like local heating by Neel relaxation of a super paramagnetic nanoparticle under an alternative magnetic field, overcome these constrains. The vicinity of the nanoparticles becomes much hotter than the macroscopic solution under the AMF and cools down to the temperature of the ambient fluid on a time scale of seconds after the magnetic field is turned off [8]. For in vitro diagnosis purposes, the size of the nanoparticles must be balanced between a high surface to volume ratio and a high magnetization.

Size and shape of nanoparticles are key parameters to determine their colloidal stability. Size has a strong impact on the magnetic moment and on the response to an applied magnetic field. For an easy manipulation by an external field, the overall magnetization must be maximized. Ferromagnetic materials such as iron, nickel or cobalt and their ferrimagnetic oxides are thus favored. The iron oxide is particularly popular under its maghemite γ-Fe_2_O_3_ form that can be synthesized with the best chemical stability against oxidation and possesses interesting electro-magnetic properties. A noteworthy surface functionalization of those magnetic cores is allowed with amorphous silica shells via the sol-gel process. The silica coating, hydrophilic, is stable in biological media (pH < 9) and can be easily functionalized with chemical groups interesting for coupling with biomolecules. Thus, such silica coated magnetic nanoparticles have been widely studied for biomedical applications, like multimodal imaging and targeted drug delivery [9]. Chemical functions charged at pH 7.4 (amine, carboxylic acid) and polymer PEG chains bring electrostatic and steric repulsions ensuring high colloidal stability in biological media [10] Moreover, another advantage of a silica layer is to limit dipolar interactions between the magnetic particles [11]. Polydopamine coating is an alternative strategy also studied, showing promising results, notably to combine magnetic resonance imaging and photothermal therapy, due to the good photothermal effect of polydopamine [12].

In this work, an easy and flexible way to release hybridized DNA targets grafted on super paramagnetic core-shell nanoparticles with efficiency using magnetic hyperthermia is presented. Our objective is not to create the most powerful nanoheaters but to achieve a robust and reproducible way to elaborate a nanoplatform with flexible properties both magnetic and biologic in aqueous solution. This strategy is simple yet efficient regarding the magnetic hyperthermia properties of the particles. To this goal, core-shell nanoparticles with a maghemite γ-Fe_2_O_3_ core and a silica shell were synthetized and functionalized with DNA on their surface for capture and release of specific microRNA.

## 2. Materials and Methods

### 2.1. Chemicals

*N*-(3-Dimethylaminopropyl)-*N*-ethylcarbodiimide hydrochloride (EDC); *N*-Hydroxy-sulfosuccinimide sodium salt (NHS); 3-(*N*-Morpholino)propanesulfonic acid sodium salt (MOPS); tetraethoxyorthosilicate (TEOS); and 3-(aminopropyl)triethoxysilane (APTS) were purchased from Sigma-Aldrich (St Quentin Fallavier, France). Citric acid was purchased from Merck (Nogent sur Marne, France). 2-[Methoxy(polyethyleneoxy)propyl]-trimethoxysilane (PEOS), containing 3-6 ethylene oxide groups, was purchased from Gelest (Morrisville, PA, USA). 30% H_2_O_2_, HCl and NaOH solutions were obtained from VWR (Strasbourg, France). The oligonucleotides were purchased from Integrated DNA Technology (Leuven, Belgium). The probe was 21-basis long and was modified with a carboxy-end for conjugation to silica coated magnetic nanoparticles. The target was an unmodified DNA sequence of 20 bases as written in Table 1 mimicking the fragment of interest, miRNA-122, for the diagnosis of liver cells in case of injury (hepatitis, alcoholism, obesity).

### 2.2. Synthesis of γ-Fe_2_O_3_@SiO_2_ Core-Shell Nanoparticles

#### 2.2.1. Synthesis of γ-Fe_2_O_3_ Nanoparticles

Maghemite γ-Fe_3_O_4_ nanoparticles (MNP) were synthesized following the Massart co-precipitation synthesis [13]. First, 180 g FeCl_2_ were weighed with 100 mL of 37% HCl and 500 mL of distilled water. A volume of 715 mL of 35.2% FeCl_3_ was added to the latter preparation with 3 L of distilled water under agitation. A volume of 1 L of 22.5% NH_3_ was then added. The black precipitate was then washed with distilled water under magnetic decantation. A volume of 360 mL of 52.5% HNO_3_ was added to redisperse magnetite nanoparticles into acidic medium. Then, in order to oxidize them in maghemite nanoparticles, 323 g of Fe(NO_3_)_3_ were weighed and added in 800 mL of water. The mix was brought to boil for 30 min. A volume of 360 mL 52.5% HNO_3_ was used first to wash the NPs, followed by three washing steps with acetone and two with ether. The final nanoparticles were dispersed in 1 L of ultra-pure water. To size-sort the biggest maghemite nanoparticles, 5 mL of 68% HNO_3_ were added to induce their destabilization [14]. The dense phase (flocculate) with the bigger particles was separated from the supernatant by magnetic decantation. The flocculate phase was kept and this size sorting step was repeated with 5 mL then with 3 mL of HNO_3_.

To stabilize the maghemite nanoparticles by electrostatic repulsion in aqueous medium at pH 7, these nanoparticles were citrated to bring negative charges at their surface. In brief, citrate with a 0.13 ratio against number of iron moles was added. The mix was heated at 80 °C for 30 min and washed with magnetic decantation using acetone and ether. The obtained nanoparticles were dispersed, as a final step, in ultra-pure water.

#### 2.2.2. Silica Coating Process

γ-Fe_2_O_3_ core-shell nanoparticles (CS A) were synthesized using a procedure developed and optimized by the PHENIX laboratory [15,16,17]. A volume of 700 µL of size sorted citrated maghemite nanoparticles ([Fe] = 1.02 M) was diluted in 50 mL of water and 100 mL of ethanol. A volume of 900 µL of TEOS and 1.88 mL of 30% NH_3_ were then added and left to react under stirring for two hours. For PEG/NH_2_ functionalization, 300 µL of TEOS, 350 µL of PEOS and 150 µL of APTS were added and left to react overnight. The obtained nanoparticles were washed with ethanol and ether with a 15:1 ratio and dispersed in 50 mL of 0.1 M MOPS buffer solution (pH 7.4).

To obtain elongated γ-Fe_2_O_3_ core-shell nanoparticles (CS B), NH_4_Cl ([NH_4_Cl] = 4 mM) was added after dilution of maghemite nanoparticles in water and ethanol to cause their slight aggregation by screening the electrostatic repulsions. Then, the silica coating and redispersion were performed as described above in the synthesis of CS A particles.

### 2.3. DNA Duplexes Conjugation on γ-Fe_2_O_3_@SiO_2_ Core-Shell Nanoparticles

To optimize the DNA-probe functionalization on MNPs, a double-strand protocol was chosen. The available commercial DNA probes have been modified with a carboxy-end for conjugation to amino-end functions on nanoparticles thanks to the well-known peptidic EDC/NHS coupling protocol. First, 10 equivalents of the target DNA were put to hybridize with 1 equivalent of its complementary probe during 30 min in 0.1 M MOPS buffer solution (pH 7.4) with 0.5 M NaCl to obtain the DNA duplexes. Then, 140 equivalents of EDC and 230 equivalents of NHS were poured into the solution for 20 min to activate the carboxyl functions on DNA duplexes. Finally, nanoparticles (78 µL of CS A or CS B in 0.1 M MOPS buffer solution) were added and left to react for 3 h. Separation between DNA duplexes attached on nanoparticles and free DNA duplexes was done using Miltenyi MS Columns by washing twice with MOPS and NaCl.

### 2.4. Characterization of γ-Fe_2_O_3_@SiO_2_ Core-Shell Nanoparticles

**Iron concentration.** Atomic adsorption spectrometry was performed to assess the iron concentration. The samples were left to incubate in 37% HCl overnight and diluted in distilled water.

**Transmission Electron Microscopy**. TEM images were obtained after placing a single drop (6 µL) diluted 1/1000 of the aqueous solution of nanoparticles onto a copper grid coated with a carbon film. The grid was left to dry in air for several hours at room temperature. TEM analysis was carried out on a JEOL 100cx electron microscope model working at 100 keV.

**Magnetostatic Superconducting QUantum Interference Device (SQUID) measurements**. Magnetostatic measurements were performed on a Quantum Design MPMS5S SQUID magnetometer. SQUID measurements were performed on dehydrated powders (dehydration in an oven at 70 °C).

**Alternating Magnetic Field assays.** The AMF assays were performed on a Magnetherm from Nanotherics with a frequency of 535 kHz and a field of 10.56 kA m^−1^. The nanoparticles solution (V = 500 µL) was introduced inside a magnetizing copper coil, which is part of a resonant RLC circuit producing an AC magnetic field in the frequency range of 300–550 kHz and with amplitudes up to 10.56 kA m^−1^. The coil was cooled down by a water circulation to get rid of the influence of thermal gradients. The bulk solution temperature was measured with a non-metallic optical fibre (Fluoroptic, Luxtron Corp., Santa Clara, CA, USA) placed in the center of the sample.

### 2.5. Fluorescence Measurements

**Figure A1:**
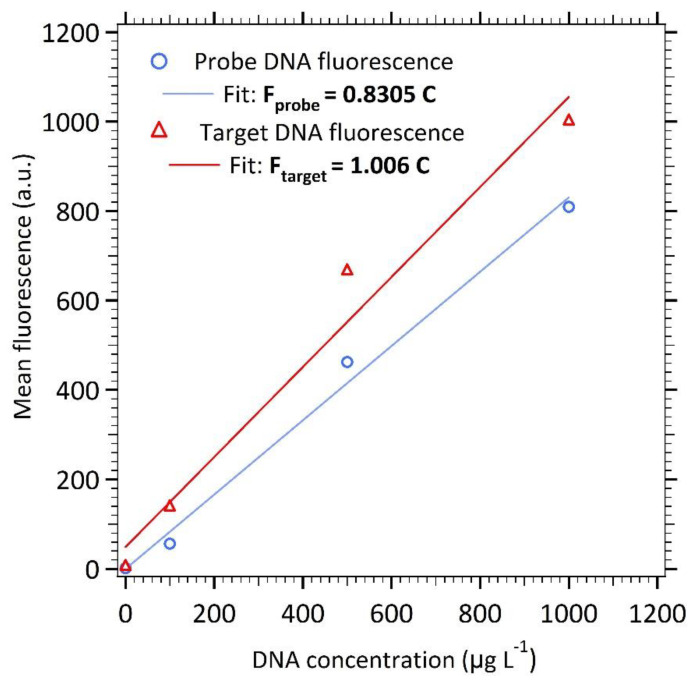
Linear calibration of the oligonucleotides using the Quant-iT Oligreen ssDNA Assay Kit.

All DNA quantification measurements were done using fluorescence spectroscopy performed on a Cary Eclipse fluorimeter with the Quant-iT^TM^ Oligreen ssDNA Assay Kit from Invitrogen [18]. The calibration curves of Quant-iT^TM^ Oligreen Assay Kit were realized with standards of DNA for the probe and the target. Samples in 4 mL cuvettes were excited at 480 nm. The fluorescence emission intensity was measured at 520 nm and plotted as a function of the DNA concentration. A linear increase in fluorescence intensity as a function of concentration is observed for both DNA types, with a higher slope for the target DNA than for the probe one (see Appendix A, Figure A1).

## 3. Results and Discussion

### 3.1. Characterization of γ-Fe_2_O_3_@SiO_2_ Core-Shell Nanoparticles

Batches of core-shell nanoparticles with an iron concentration of approximatively 15 mM were obtained from the synthesis described above. The use of such nanoparticles for recruitment of oligonucleotides in an in-vitro diagnostic test is driven by their morphology, their colloidal stability and their magnetic properties. Once our core-shell nanoparticles were fully characterized with Transmission Electron Spectroscopy (TEM) and Supra Quantum Interferometric Device (SQUID), their heating efficiency were assessed with magnetic hyperthermia.

#### 3.1.1. From a Spherical to a Rod-Like Morphology

Magnetic nanoparticles are not entirely monodisperse and a log-normal size distribution (*P*) as written in Equation (1) is usually assumed [19]. The mean physical diameter (*d_phys_*) of nanoparticles and polydispersity index (*σ*) can be extracted from the log-normal distribution, as follows

(1) P(d)=12π·σ·d·e−ln(dd0)22σ2

and *σ* is related to a maximal value ln(*d_phys_*) by:

(2) $$d_{phys}=\;d_{0}\cdot e^{\sigma^{-2}}$$

The histogram *P*(*d*) of the size distribution was obtained by counting nanoparticles with sizes within a given size bin on TEM images using the Image J software on approximately 200 particles. Size histograms were then fitted according to a log-normal distribution.

**Figure 1:**
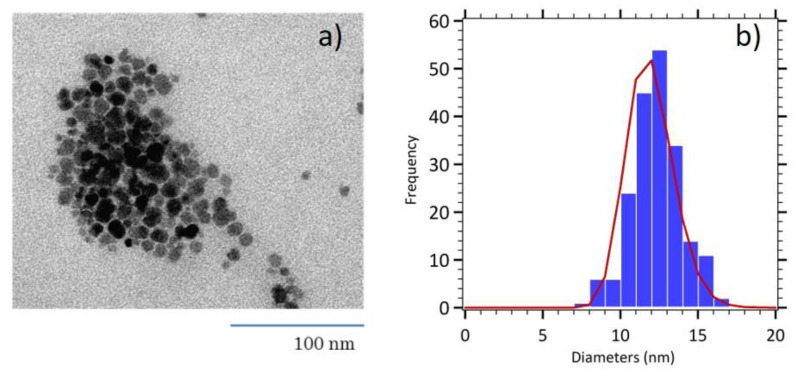
(**a**) TEM image of size sorted maghemite nanoparticles (MNP). (**b**) Size histogram fitted with the log normal distribution (red line).

To obtain more efficient nanoparticles, regarding their magnetic properties, maghemite nanoparticles were size sorted to retain only the biggest nanoparticles and to decrease the polydispersity index *σ*. Indeed, especially for magnetic hyperthermia, it has been observed that size dispersion strongly weakens the heating efficiency of samples between 12 and 17 nm diameters [20]. Size selection with nitric acid to induce destabilization is causing larger particles to precipitate and leaving smaller and nearly monodisperse particles in the supernatant. The TEM image on Figure 1 displays nanoparticles of poorly controlled, roughly spherical shape. The size histogram shows a narrow distribution with a mean physical diameter of 12 nm (*σ* = 0.12) indicating the success of our size sorting procedure.

**Figure 2:**
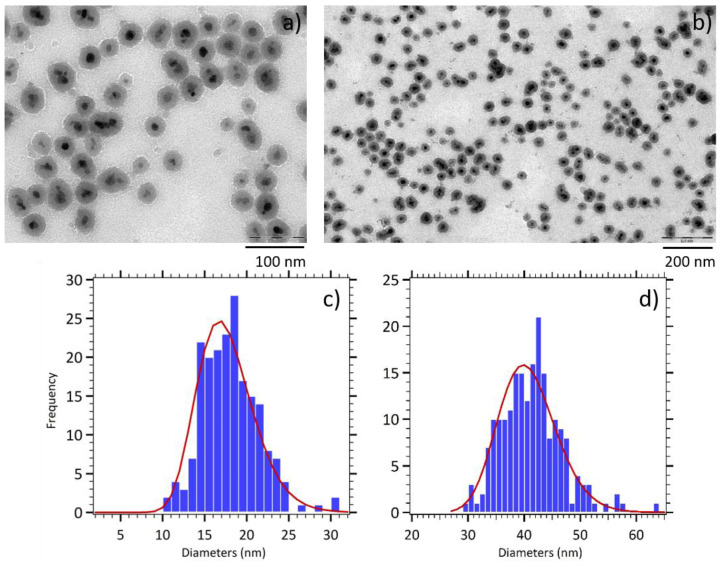
(**a**,**b**) TEM images of γ-Fe_2_O_3_@SiO_2_ core-shell nanoparticles (CS A). Size histograms fitted with the log normal distribution (red line) for (**c**) magnetic cores and (**d**) entire nanoparticles.

The silica coating of maghemite nanoparticles produces composite nanoparticles made of a magnetic core coated by a silica shell. As shown by the TEM images reported in Figure 2, the γ-Fe_2_O_3_@SiO_2_ core-shell nanoparticles (CS A) were roughly spherical, with a magnetic core formed by a few maghemite nanoparticles. Size distributions analysis reveal a mean physical diameter of 17 nm (*σ* = 0.18) for magnetic cores and of 40 nm (*σ* = 0.13) for the entire nanoparticles. This implies a silica shell thickness of approximately 10 nm, as can be observed in the TEM images.

**Figure 3:**
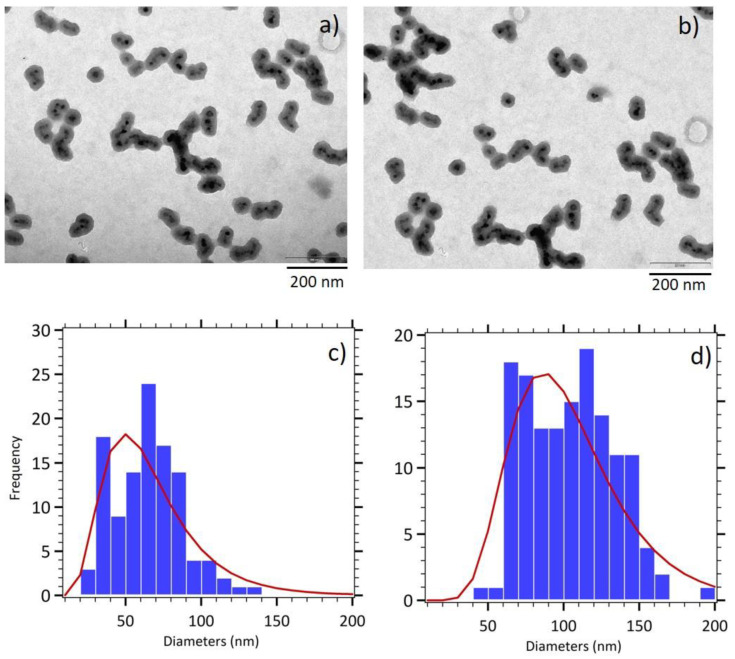
(**a**,**b**) TEM images of elongated γ-Fe_2_O_3_@SiO_2_ core-shell nanoparticles (CS B). Size histograms fitted with the log normal distribution (red line) for (**c**) magnetic cores and (**d**) entire nanoparticles.

Pre-aggregation of maghemite cores in the reaction media was performed adding NH_4_Cl before the silica shell growth. The increase of the ionic strength allows to screen electrostatic repulsions of the negative charges at the surface of maghemite nanoparticles, brought by the citrate adsorbed molecules, thus leading to a nanocluster formation. As expected, the addition of a controlled amount of NH_4_Cl allows composite nanoparticles of a larger size to be obtained, the magnetic core being composed of a larger number of maghemite nanoparticles (Figure 3a,b). Moreover, a shift from a spherical morphology to a rod like morphology is observed where maghemite sorted cores form short chain-like structures inside the silica shell. This property is attributed to an alignment of the magnetic domains during the nanocluster formation, due to the strong magnetic dipole interactions for the used large diameter maghemite nanoparticles. The determination of the length of the magnetic cores (60 nm, *σ* = 0.44) and of the silica shell (98 nm, *σ* = 0.35) thanks to size distribution analysis confirmed the increased size of those elongated γ-Fe_2_O_3_@SiO_2_ core-shell nanoparticles (CS B) and a broader size distribution (Figure 3c,d).

#### 3.1.2. Magnetostatic Properties

**Figure 4:**
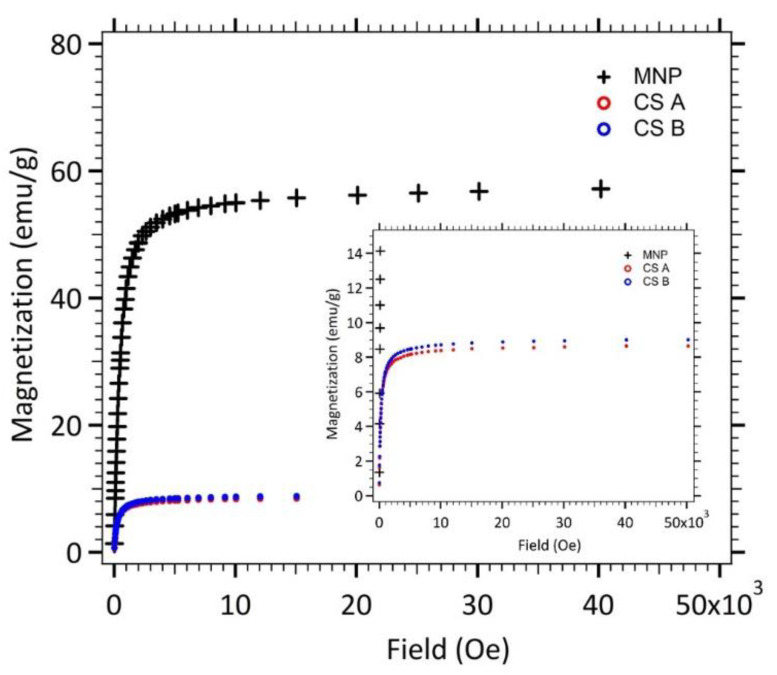
Magnetization of nanoparticles (MNP, CS A and B) in powder state versus the applied magnetic field.

Figure 4 displays the magnetostatic signature of maghemite nanoparticles (MNP), spherical γ-Fe_2_O_3_@SiO_2_ core-shell nanoparticles (CS A) and elongated ones (CS B). The saturation magnetization *M_sat_* (i.e., the maximum induced magnetic moment that can be obtained in a magnetic field) is equal to 56.5 emu/g for maghemite nanoparticles (MNP). The saturation magnetization of bulk maghemite, 85 emu/g, is not reached, which reveals that the magnetic order is imperfect in each maghemite nanoparticles. This reduced magnetization is often observed for nanoparticles and generally attributed to a non-magnetic surface layer (surface effect disorders) [20].

Magnetization measurements for core-shell nanoparticles lead to smaller saturation magnetization values as expected for composite nanoparticles. The *M_sat_* values of both kind of core-shell nanoparticles (CS A and B) was divided by the saturation magnetization of the maghemite nanoparticles, leading to the mass ratio of maghemite.

The fit of the theoretical expression (Langevin formalism [21]) to the experimental magnetization curve (for dilute solution samples—data not shown), weighted by the size distribution, allowed the determination of the characteristic magnetic diameter, *d_mag_*, and the polydispersity index, *σ*, for each sample. The mean value of the magnetic diameters can be calculated in order to consider the polydispersity with the following Equation (3),

(3) m¯= dmag·e− σ22

The obtained values, quite similar for spherical and elongated core shell nanoparticles are reported in Table 2.

The calculated $\bar{m}$ parameter is found to be roughly the same and independent of the particle’s size. The *σ* values of polydispersity in magnetic domains are consistent with the values of standard home-made ferrofluids which polydispersity indexes are around 0.35–0.4 [20].

#### 3.1.3. Heating Efficiency

In brief, under a radiofrequency alternating magnetic field (AMF), the magnetic moments of iron oxide nano-crystals oscillate and produce a local heating of the medium [22]. The AMF induces a dephasing between the applied field and the magnetic momentum of the nanoparticle’s domain yielding dynamic hysteresis. The excited magnetic dipoles release energy in the form of heat while relaxing. The heating effects are due to several types of loss processes (hysteresis losses, Neel and Brown relaxation), the relative contributions of which depend strongly on particle size. Nanoparticles with a core diameter less than 30 nm are supposed to be single-domain particles. Their magnetization is thus governed by the combined effects of the rotational external (Brownian) and internal (Neel) diffusion of the particle magnetic momentum [23].

**Figure 5:**
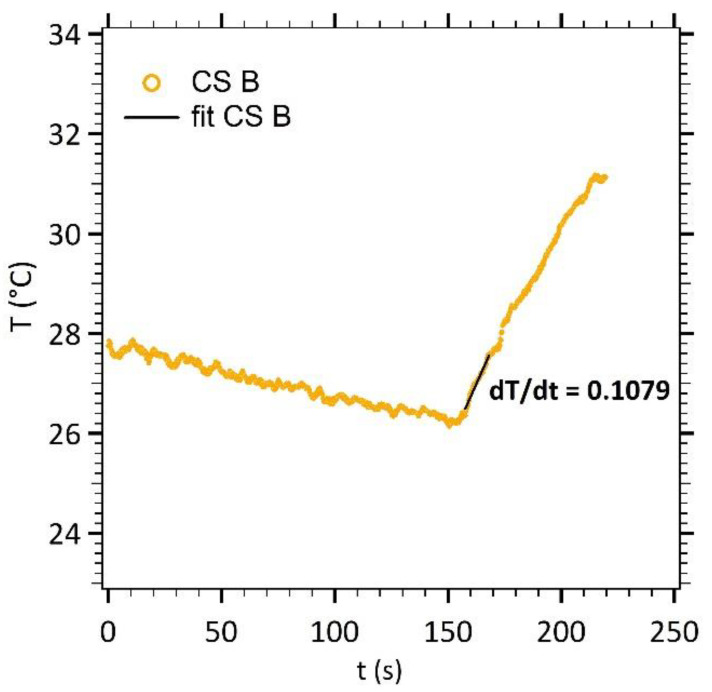
Temperature measurements as a function of time (CS B).

To characterize the effect of the core diameter increase on the heating efficiency of our nanoparticles, a magnetic field signal (535 kHz and 10.56 kA m^−1^) was applied for a few minutes in concentrated solutions. For instance, Figure 5 displays the increase in temperature for elongated γ-Fe_2_O_3_@SiO_2_ core-shell nanoparticles (CS B).

The temperature increase was approximated by a linear variation as a function of time. The *dT*/*dt* experimental slope (straight line) was calculated on a 13 s linear portion of the increasing temperature (50 points of measure, initial slope method [24]). The Specific Loss Power (SLP) of the nanoparticles was then calculated according to Equation (4).

(4) $$SLP=\frac{m_{total}}{m_{mag}}\cdot c_{p}\cdot \frac{dT}{dt}$$

with *c_p_* the specific heat capacity of water, *m_total_* the total mass of the sample and *m_mag_* the mass of magnetic material.

To be able to compare our results with other previously reported results, where AMF setups use different working frequency *f* and/or magnetic field magnitude *H*, the SLP was normalized to give the Intrinsic Loss Power (ILP) formulation, and was expressed according to Equation (5), as follows

(5) $$ILP=\;\frac{SLP}{H^{2}\cdot f}$$

with, *H* the amplitude of the alternating magnetic field and *f* its frequency.

Table 3 gathers the *dT*/*dt* experimental slopes, SLP and ILP values for maghemite nanoparticles (MNP), spherical γ-Fe_2_O_3_@SiO_2_ core-shell nanoparticles (CS A) and elongated ones (CS B). The ILP values of our home-made nanoparticles are around the same order of magnitude of the commercial ones [25]. A slight increase of the SLP and ILP values is observed for the elongated γ-Fe_2_O_3_@SiO_2_ core-shell nanoparticles (CS B) in comparison to the spherical ones (CS A).

Our particles are composed of multiple single domain maghemite cores aggregated inside a silica shell forming short chain-like aggregates. As discussed previously, in a liquid medium, both Neel and Brown relaxation occur, the dominant process being the one with the shortest characteristic time (1/τ = 1/τ_N_ + 1/τ_B_). For a monodomain core, the time necessary to jump over the magnetic anisotropy energy barrier K.V is governed by the Neel relaxation time with τ_N_ in the order of magnitude of a few nanoseconds. For bigger particles, there is a contribution of the Brown relaxation. Effective anisotropy is tunable through shape and surface modification. Due to the limited heating capacities of spherical magnetite or maghemite, Das et al. designed Fe_3_O_4_ nanorods and reported a 0.68 m^2^ nH kg^−1^ [26] ILP value, higher than for nanoparticles with similar volume (0.11 m^2^ nH kg^−1^ for spheres, 0.25 m^2^ nH kg^−1^ for cubes). Their work pinpoints that the ILP of iron oxides can be increased and tuned by acting on their aspect ratio. The latter point is crucial even if volumes are similar, which is not obvious in Neel’s description. Martinez-Boubeta et al. reported that chainlike formation for nanocubes [27] or biomimicking magnetostactic bacteria yielded increased heating efficiencies compared to randomly oriented systems up to 2.7 m^2^ nH kg^−1^.

For short chain-like structures, the increase in heating efficiency can be ascribed to two contributions. A major contribution is from the chain geometry: the hysteresis loop is more rectangular for a chain than for a sphere [28]. The minor contribution comes from texturization effect. The released energy by the dipole relaxation is dependent on the anisotropy energy of the nanoparticle. The latter imposes the direction of the magnetic momentum against the crystalline network. The magnetic energy of a chain is dominated by anisotropic dipole-dipole interactions which favors longitudinal alignment of the magnet along crystalline axes during the chain formation. If the nanoparticle magnetic anisotropy axes are aligned, parallel to the chain axis, the effective anisotropy is higher due to a magnetic order. Although these predictions cannot be strictly extrapolated to our case as magnetic cores are randomly brought together, the existence of a certain magnetic order between chains should not be disregarded.

### 3.2. DNA Release in Magnetic Hyperthermia

Prior to the use of γ-Fe_2_O_3_@SiO_2_ core-shell nanoparticles in a diagnostic application to detect miRNA-122, the release of grafted double stranded DNA was studied.

**Figure 6:**
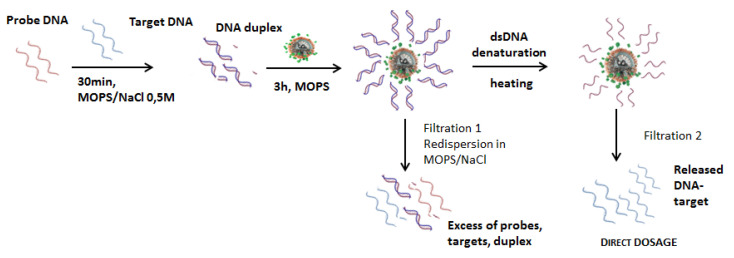
Double strand DNA grafting protocol and subsequent DNA target fluorescence detection after heating.

While a lot of -NH_2_ groups are theoretically available on the nanoparticles surface, the carboxyl-modified DNA probes cannot all react due to steric hindrance. The number of DNA probes possible to couple will be far below the number of amine groups available at the surface. Moreover, to reach the highest efficiency for later hybridization with complementary DNA target, like miRNA-122, at room temperature, DNA probes should not be too densely packed. Indeed, both strands are negatively charged, and enough space should be left for the target to come near the probe and to hybridize at the surface. In order to validate the DNA target release protocol, the optimization of a double strand protocol performances was thus first studied, as summarized in Figure 6.

To roughly get an order of magnitude of oligonucleotides’ quantities to attach, the ratio between a CS A nanoparticle’s surface and the basis of the double helix formed by double-stranded DNA was estimated approximately equal to 400. The conjugation protocol with CS A nanoparticles was then performed using a large excess of DNA probes, around 1400 per core shell nanoparticles, 10 equivalents of DNA target per DNA probe being incubated at the first step of the protocol. For CS B nanoparticles, taking into consideration the larger size and the rod-like shape, 2500 DNA probes per core shell nanoparticles were introduced in the conjugation protocol, which corresponds to about 3.5 times the maximum amount estimated by comparing the surfaces.

Once formed, the DNA duplexes were grafted via the carboxy-modified end of the probe sequence, thanks to carbodiimide activation of the carboxylic acid group. Excess DNA probes and targets were eliminated with magnetic separation and the biofunctionalized core shell nanoparticles were redispersed in MOPS buffer solution. Using such large excess might allow to saturate the surface of nanoparticles with double stranded DNA. The number of grafted DNA targets was assessed, by fluorescence spectroscopy, after dissociation from their probe, either by global heating or by local heating, with magnetic hyperthermia.

DNA strands are attached in a reversibly way by hydrogen bounds between nucleotides. The melting temperature (*Tm*) is defined as the temperature when half of the DNA targets are dissociated from their complementary probes. If *T* > *Tm*, the hydrogen bounds break abruptly as it is a reaction from a cooperative nature. Due to the dissociation-characteristics of double-stranded DNA during heating, the energy required to break the base-base-hydrogen bonding between two strands of DNA is dependent on several parameters. *Tm* is given by the Equation (6), as follows

(6) $$T_{m}=16.6\log\left[Na^{+}\right]+0.41\left(\%GC\right)+81.5-\frac{675}{l_{\mathrm{basis}}}$$

where *l*_basis_ is the base pair length, [Na^+^] represents the buffer saline concentration of the medium, and %*GC* indicates the composition in *G* and *C* bases in the sequence [29].

In this work, a target of 21 bases (7.14 nm in length), a saline buffer concentration of 0.5 M and a *GC* composition of 47.6%, were used, leading to a melting temperature of around 55 °C. Nevertheless, the melting temperature of immobilized double stranded DNA tends to be higher than melting temperature of DNA in solution [30] as the strands are less accessible. According to the calculations of Dias et al. [3], the melting temperature on the surface should approach the melting temperature in solution by 5 to 8 °C.

**Figure 7:**
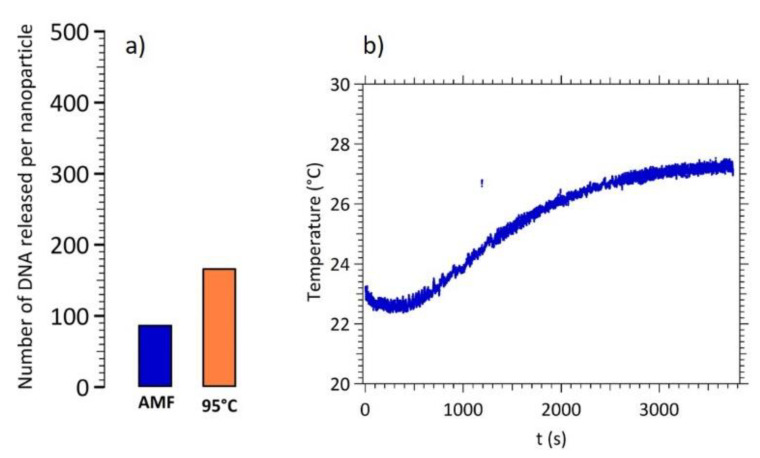
(**a**) Number of released DNA targets per CS A nanoparticles by magnetic hyperthermia and by global heating at 95 °C. (**b**) Temperature monitoring during the magnetic hyperthermia experiment.

As described previously, the major advantage of DNA target dissociation using magnetic hyperthermia is the local heating in the surroundings of the particle (e.g., a few nanometers [3]) while leaving the solution at room temperature. Figure 7 shows a comparison between the number of DNA target per CS A nanoparticles released by a global heating at 95 °C and by magnetic hyperthermia (layout a). One can note that magnetic hyperthermia is only about half efficient than a global heating while keeping the temperature close to the initial one (layout b).

**Figure 8:**
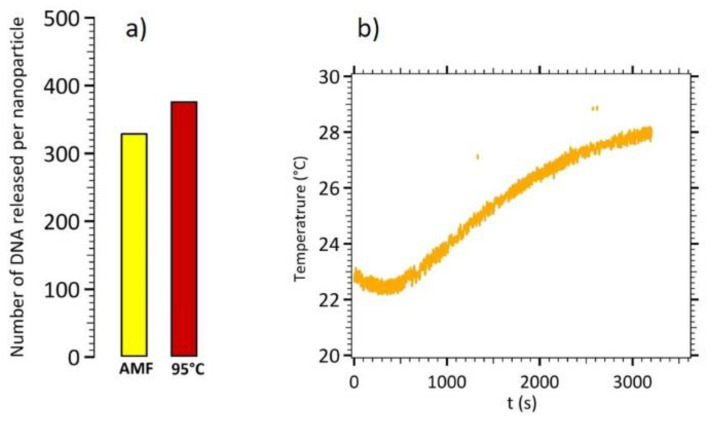
(**a**) Number of released DNA targets per CS B nanoparticles by magnetic hyperthermia and by global heating at 95 °C. (**b**) Temperature monitoring during the magnetic hyperthermia experiment.

The same experiment was performed for CS B nanoparticles. Figure 8 displays the experimental results for DNA release using magnetic hyperthermia on CS B and its comparison with a global heating at 95 °C.

In these experiments, a time of 20 min for 95 °C global heating was set as the reference experiment, corresponding to 100% of DNA target released from the nanoparticles’ surface. Around 333 DNA per CS B nanoparticles were released in 1 h of magnetic hyperthermia, without significant increase of the temperature, leading to 88% efficiency in comparison with global heating at 95 °C.

**Figure A2:**
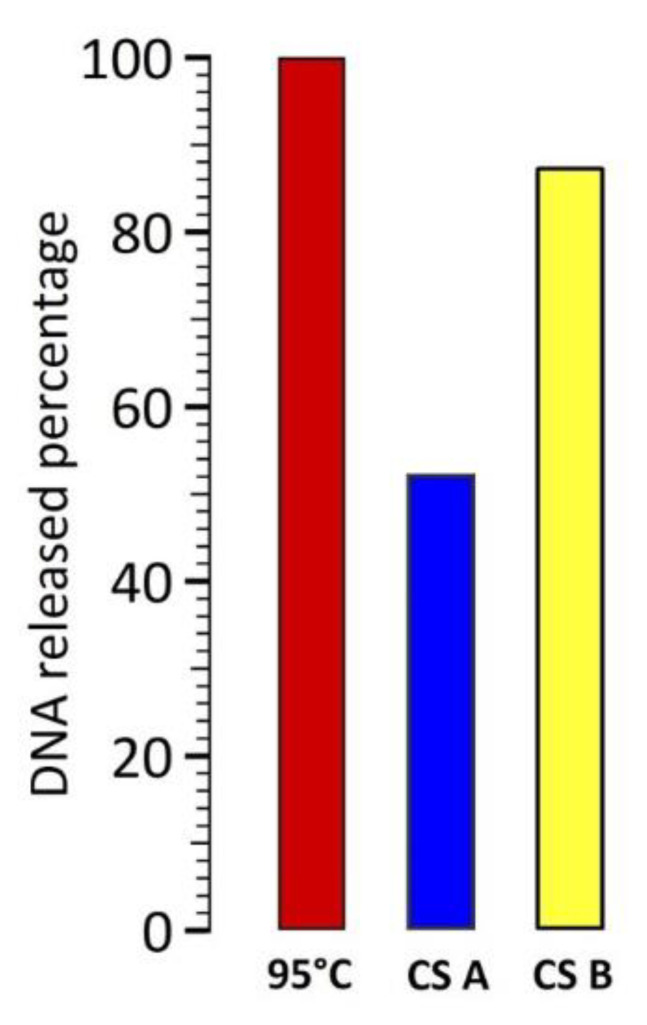
DNA target release efficiencies calculated after estimation of the total number of DNA released when the samples were heated up to 95 °C for 20 min (red histogram as reference).

Local heating on CS nanoparticles B and A was 88% and 52% as efficient as a global heating at 95 °C, respectively (see Appendix B, Figure A2), with no significant increase of the temperature. The main advantage lies in terms of temperature control, as no feedback loop will be needed and the integrity of temperature sensitive components will be preserved, in a future lab on chip dedicated to diagnosis. Magnetic hyperthermia on CS B results in higher DNA release efficiency due to improved magnetic hyperthermia performance, related to their short-chain like structures.

## 4. Conclusions

The synthesized γ-Fe_2_O_3_@SiO_2_ core-shell nanoparticles, stable at biological pH, bears novel and tunable properties. In this work, a protocol for a slight aggregation of magnetic cores before silica coating that lead to nanoparticle shape modification, from a spherical to a rod-like morphology, was proposed. Those elongated γ-Fe_2_O_3_@SiO_2_ core-shell nanoparticles displayed larger size and interesting magnetic properties for magnetic hyperthermia. The increase of SLP can be explained by considering the magnetic order inside a nanoparticle when the morphology is shifting from a spherical to a rod-like one. These synthesized core-shell nanoparticles were used to conjugate DNA probes hybridized with their complementary DNA targets mimicking the liver specific oncotarget miRNA-122. The subsequent dissociation of DNA targets with a global heating (95 °C) was successful to release up to around 400 DNA targets per nanoparticle. Magnetic hyperthermia experiments showed that elongated γ-Fe_2_O_3_@SiO_2_ core-shell nanoparticles were almost as efficient in local heating as a 95 °C global heating while keeping the solution at 28 °C, crucial for biological media and energy efficiency. This work can be considered as a proof of concept for tunable DNA release at room temperature using magnetic hyperthermia. Future works will be dedicated to DNA targets capture, with synthetic DNA probes immobilized onto the nanoparticles‘ surface, within real biological samples containing different sequences of microRNA in plasma or in saliva for early diagnosis.

## Figures and Tables

**Table 1:** Sequences of oligonucleotide strands.

Oligonucleotide	Sequence (5′ to 3′)
Probe (P)	5′-Carboxy C6-CAA ACA CCA TTG TCA CAC TGC-3′
Target (T)	5′-GC AGT GTG ACA ATG GTG TTT G-3′

**Table 2:** Magnetic diameters and magnetic materials ratio.

	*d_mag_* (nm)	σ	$\bar{m}\;(\mathrm{nm})$	$M_{sat}^{}\;(\mathrm{emu}/g)$	Maghemite Mass Ratio (%)
MNP	10.9	0.37	11.7	56.5	N/A
CS A	9.4	0.30	9.83	8.63	15.3
CS B	8.5	0.41	9.25	8.99	15.9

**Table 3:** Parameters comparison between nanoparticles synthetized in this work and commercial ones.

Particles	$\bar{m}$ (nm) ^†^	[Fe_2_O_3_] (mg·mL^−1^)	dT/dt (°C·s^−1^)	SLP (W·g^−1^)	ILP (m^2^·nH·kg^−1^)
MNP	11.7	6.16	0.077	52	0.88
CS A	9.83	5.60	0.085	64	1.06
CS B	9.25	5.36	0.105	82	1.37

^†^ Mean magnetic diameter.

## Data Availability

Data sharing is not applicable to this article.
